# Supplementary material for: Machine Learning Accelerates Crystallization for Structure Determination
Source: Angew Chem Int Ed Engl. 2026 May 4;65(25):e1218503. doi: 10.1002/anie.1218503 (PMC13266958; doi:10.1002/anie.1218503)

checkCIF/PLATON report

Structure factors have been supplied for datablock(s) lcz091

THIS REPORT IS FOR GUIDANCE ONLY. IF USED AS PART OF A REVIEW PROCEDURE FOR PUBLICATION, IT SHOULD NOT REPLACE THE EXPERTISE OF AN EXPERIENCED CRYSTALLOGRAPHIC REFEREE.

No syntax errors found. CIF dictionary Interpreting this report

Datablock: lcz091

Bond precision:	C-C = 0.0194 A		Wavelength=1.54184
Cell:	a=9.0851(1)	b=18.7880(2)	c=20.9519(2)
	alpha=90	beta=93.282(1)	gamma=90
Temperature:	100 K		

	Calculated	Reported
Volume	3570.43(6)	3570.43(6)
Space group	P 21	P 1 21 1
Hall group	P 2yb	P 2yb
Moiety formula	C28 H14 Ag3 F18 N7 S2	0.5(C15 H3 Ag3 F18 N6), C14 H7 Ag1.5 F9 N3.5 S, 0.5(C13 H11 N S
Sum formula	C28 H14 Ag3 F18 N7 S2	C28 H14 Ag3 F18 N7 S2
Mr	1178.19	1178.19
Dx,g cm-3	2.192	2.192
Z	4	4
Mu (mm-1)	15.375	15.375
F000	2264.0	2264.0
F000’	2276.88	
h,k,lmax	11,23,26	11,23,26
Nref	15369[7926]	11667
Tmin,Tmax	0.198,0.215	0.196,1.000
Tmin’	0.037	

Correction method= # Reported T Limits: Tmin=0.196 Tmax=1.000
AbsCorr = MULTI-SCAN

Data completeness= 1.47/0.76   Theta(max)= 78.458

R(reflections)= 0.0462(11031)   wR2(reflections)= 0.1123(11667)

S = 1.096   Npar= 1048

The following ALERTS were generated. Each ALERT has the format
test-name_ALERT_alert-type_alert-level.
Click on the hyperlinks for more details of the test.

Alert level C

```
PLAT042_ALERT_1_C Calc. and Reported MoietyFormula Strings  Differ     Please Check
              Calc: C28 H14 Ag3 F18 N7 S2
              Rep.: 0.5(C15 H3 Ag3 F18 N6), C14 H7 Ag1.5 F9 N3.5 S
                  , 0.5(C13 H11 N S2)
PLAT090_ALERT_3_C Poor Data / Parameter Ratio (Zmax > 18) ........       7.22 Note
PLAT213_ALERT_2_C Atom F02R            has ADP max/min Ratio .....        3.7 prolat
PLAT220_ALERT_2_C NonSolvent   Resd 1  C   Ueq(max)/Ueq(min) Range        3.2 Ratio
PLAT220_ALERT_2_C NonSolvent   Resd 1  F   Ueq(max)/Ueq(min) Range        4.1 Ratio
PLAT342_ALERT_3_C Low Bond Precision on  C-C Bonds ..............    0.01944 Ang.
PLAT601_ALERT_2_C Unit-Cell Contains Solvent Accessible VOIDS .LE.         40 Ang**3
PLAT911_ALERT_3_C Missing FCF Refl Between Thmin & STh/L=    0.600         13 Report
              -10  0  1,  10  0  1,   0  2  1,   0  0  2,  -1  1  2,   9  0  3,
               -1 22  3,   1 15  7,  -2 21  7,  -3 20  9, -10  0 11,   0  0 21,
                0  0 25,
PLAT915_ALERT_3_C No Flack x Check Done: Low Friedel Pair Coverage         55 %
PLAT934_ALERT_3_C Number of (Iobs-Icalc)/Sigma(W) > 10 Outliers ..          1 Check
               10 -7  5,
```

Alert level G

```
PLAT003_ALERT_2_G Number of Uiso or U(i,j)  Restrained non-H-Atoms         13 Report
PLAT004_ALERT_5_G Polymeric Structure Found with Maximum Dimension          1 Info
PLAT007_ALERT_5_G Number of Unrefined Donor-H Atoms .............           2 Report
              H01U  H01D
PLAT033_ALERT_4_G Flack x Value Deviates > 3.0 * Sigma from Zero .      0.157 Note
PLAT083_ALERT_2_G SHELXL Second Parameter in WGHT  Unusually Large      37.04 Why ?
PLAT143_ALERT_4_G s.u. on c - Axis Small or Missing .............    0.00020 Ang.
PLAT186_ALERT_4_G The CIF-Embedded .res File Contains ISOR Records          4 Report
PLAT242_ALERT_2_G Low    'MainMol' Ueq as Compared to Neighbors of       C020 Check
PLAT242_ALERT_2_G Low    'MainMol' Ueq as Compared to Neighbors of       C02D Check
PLAT242_ALERT_2_G Low    'MainMol' Ueq as Compared to Neighbors of       C02Q Check
PLAT242_ALERT_2_G Low    'MainMol' Ueq as Compared to Neighbors of       C02W Check
PLAT242_ALERT_2_G Low    'MainMol' Ueq as Compared to Neighbors of       C034 Check
PLAT242_ALERT_2_G Low    'MainMol' Ueq as Compared to Neighbors of       C035 Check
PLAT242_ALERT_2_G Low    'MainMol' Ueq as Compared to Neighbors of       C01Y Check
PLAT242_ALERT_2_G Low    'MainMol' Ueq as Compared to Neighbors of       C024 Check
PLAT242_ALERT_2_G Low    'MainMol' Ueq as Compared to Neighbors of       C02K Check
PLAT242_ALERT_2_G Low    'MainMol' Ueq as Compared to Neighbors of       C02Y Check
```

```
PLAT242_ALERT_2_G Low    'MainMol' Ueq as Compared to Neighbors of       C033 Check
PLAT434_ALERT_2_G Short Inter HL..HL Contact F00G     ..F00J     .       2.80 Ang.
                                               -x,1/2+y,-z  =      2_555 Check
PLAT720_ALERT_4_G Number of Unusual/Non-Standard Labels ..........        144 Note
              Ag01    Ag02    Ag03    Ag04    Ag05    Ag06    S007    S008
              S009    S00A    F00B    F00C    F00D    F00E    F00F    F00G
              F00H    F00I    F00J    F00K    F00L    F00M    F00N    F00O
              N00P    F00Q    F00R    F00S    N00T    F00U    F00V    F00W
              F00X    F00Y    N00Z    N010    N011    N012    F013    F014
              N015    N016    F017    N018    F019    N01A    N01B    N01C
              N01D    H01D    F01E    F01F    C01G    C01H    H01H    F01I
              C01J    H01J    F01K    F01L    C01M    F01N    F01O    C01P
              H01P    F01Q    C01R    C01S    C01T    H01T    N01U    H01U
              C01V    C01W    H01W    C01X    C01Y    C01Z    C020    C021
              H021    C022    H022    C023    C024    C025    H025    F026
              C027    H027    C028    C029    C02A    H02A    C02B    H02B
              C02C    C02D    C02E    C02F    H02F    C02G    C02H    C02I
              C02J    H02J    C02K    C02L    C02M    C02N    H02N    C02O
              C02P    H02P    C02Q    F02R    C02S    C02T    C02U    H02U
              C02V    H02V    C02W    C02X    C02Y    C02Z    H02Z    C030
              H030    C031    C032    C033    C034    C035    C036    H036
              C037    H03D    H03E    H03F    C038    H03A    H03B    H03C
PLAT860_ALERT_3_G Number of Least-Squares Restraints .............         79 Note
PLAT883_ALERT_1_G Absent Datum for _atom_sites_solution_primary ..     Please Do !
PLAT910_ALERT_3_G Missing FCF Reflection(s) Below Theta(Min)[Deg]=       3.16 Note
                0  0  1,
PLAT912_ALERT_4_G Missing # of FCF Reflections Above STh/L=  0.600        343 Note
PLAT933_ALERT_2_G Number of HKL-OMIT Records in Embedded .res File          4 Note
               10 -7  4,   9-11  5,  10 -8  5,  -9 -8  1,
PLAT941_ALERT_3_G Average HKL Measurement Multiplicity ..........        2.6 Low
PLAT969_ALERT_5_G The 'Henn et al.' R-Factor-gap value ..........      2.231 Note
              Predicted wR2: Based on SigI**2  5.03 or SHELX Weight 10.25
PLAT978_ALERT_2_G Number C-C Bonds with Positive Residual Density.          0 Info
```

```
   0 ALERT level A = Most likely a serious problem - resolve or explain
   0 ALERT level B = A potentially serious problem, consider carefully
  10 ALERT level C = Check. Ensure it is not caused by an omission or oversight
  28 ALERT level G = General information/check it is not something unexpected

   2 ALERT type 1 CIF construction/syntax error, inconsistent or missing data
  20 ALERT type 2 Indicator that the structure model may be wrong or deficient
   8 ALERT type 3 Indicator that the structure quality may be low
   5 ALERT type 4 Improvement, methodology, query or suggestion
   3 ALERT type 5 Informative message, check
```

Validation response form

Please find below a validation response form (VRF) that can be filled in and pasted into your CIF.

```
# start Validation Reply Form
_vrf_PLAT042_lcz091
;
PROBLEM: Calc. and Reported MoietyFormula Strings  Differ     Please Check
RESPONSE: ...
;
```

```
_vrf_PLAT090_lcz091
;
PROBLEM: Poor Data / Parameter Ratio (Zmax > 18) ........       7.22 Note
RESPONSE: ...
;
_vrf_PLAT213_lcz091
;
PROBLEM: Atom F02R            has ADP max/min Ratio .....        3.7 prolat
RESPONSE: ...
;
_vrf_PLAT220_lcz091
;
PROBLEM: NonSolvent   Resd 1  C   Ueq(max)/Ueq(min) Range        3.2 Ratio
RESPONSE: ...
;
_vrf_PLAT342_lcz091
;
PROBLEM: Low Bond Precision on  C-C Bonds ..............    0.01944 Ang.
RESPONSE: ...
;
_vrf_PLAT601_lcz091
;
PROBLEM: Unit-Cell Contains Solvent Accessible VOIDS .LE.         40 Ang**3
RESPONSE: ...
;
_vrf_PLAT911_lcz091
;
PROBLEM: Missing FCF Refl Between Thmin & STh/L=    0.600         13 Report
RESPONSE: ...
;
_vrf_PLAT915_lcz091
;
PROBLEM: No Flack x Check Done: Low Friedel Pair Coverage         55 %
RESPONSE: ...
;
_vrf_PLAT934_lcz091
;
PROBLEM: Number of (Iobs-Icalc)/Sigma(W) > 10 Outliers ..          1 Check
RESPONSE: ...
;
# end Validation Reply Form
```

It is advisable to attempt to resolve as many as possible of the alerts in all categories. Often the minor alerts point to easily fixed oversights, errors and omissions in your CIF or refinement strategy, so attention to these fine details can be worthwhile. It is up to the individual to critically assess their own results and, if necessary, seek expert advice.

PLATON version of 17/09/2025; check.def file version of 17/09/2025

---

duplicate check

No duplication found

Datablock lcz091 - ellipsoid plot

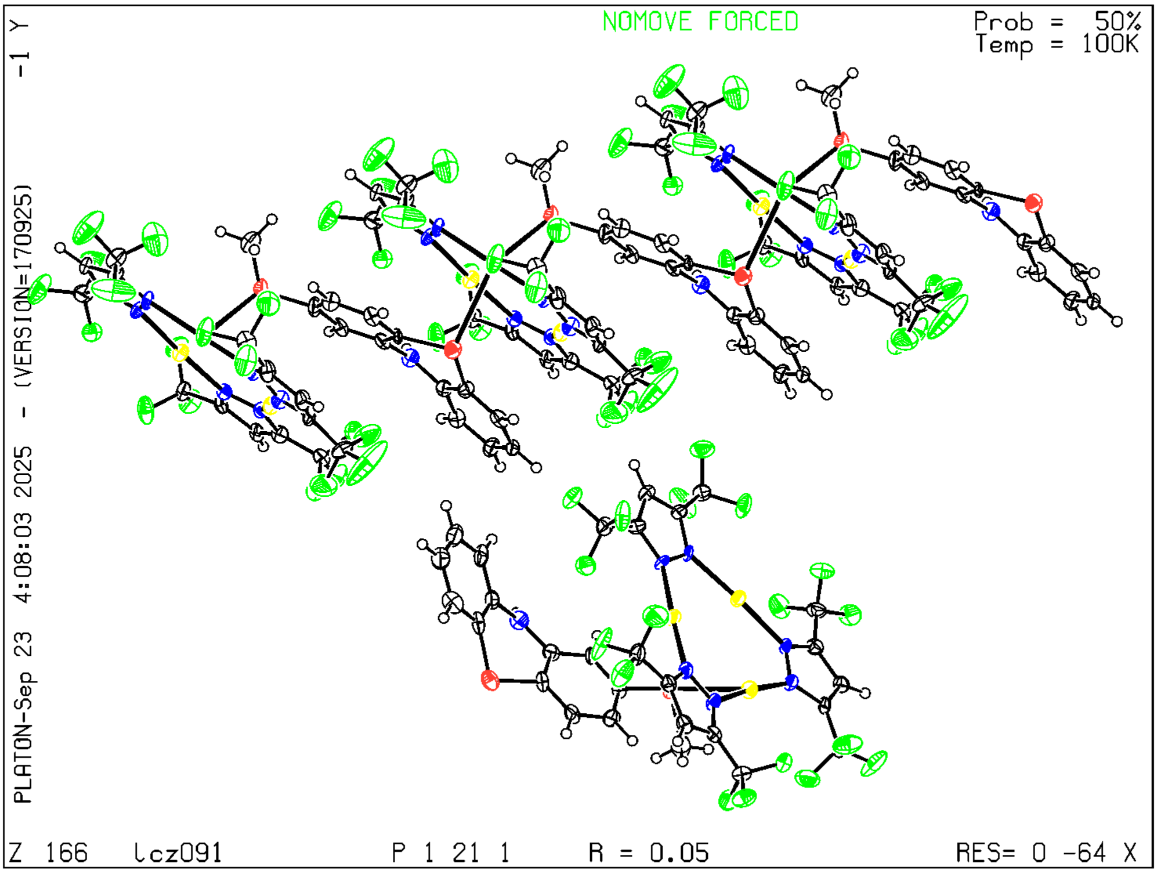

Supplement: Supplementary file 2 — Supporting File 2: anie72462‐sup‐0002‐DataFile.zip. [file ANIE-65-e1218503-s002.zip › supplementary_materials_crystallographic_data submit.zip!/cif_and_checkcif(1-114)/Ag3Pz3_91/Ag3Pz3_91_checkcif.pdf]
